# Identification of agents effective against multiple toxins and viruses by host-oriented cell targeting

**DOI:** 10.1038/srep13476

**Published:** 2015-08-27

**Authors:** Leeor Zilbermintz, William Leonardi, Sun-Young Jeong, Megan Sjodt, Ryan McComb, Chi-Lee C. Ho, Cary Retterer, Dima Gharaibeh, Rouzbeh Zamani, Veronica Soloveva, Sina Bavari, Anastasia Levitin, Joel West, Kenneth A. Bradley, Robert T. Clubb, Stanley N. Cohen, Vivek Gupta, Mikhail Martchenko

**Affiliations:** 1 Keck Graduate Institute, Claremont, CA 91711; 2 Department of Genetics, Stanford University School of Medicine, Stanford, CA 94305; 3 Department of Chemistry and Biochemistry, University of California, Los Angeles, CA, 90095; 4 US Army Medical Research Institute of Infectious Diseases (USAMRIID), Fort Detrick, MD, 21702; 5 Department of Microbiology, Immunology, and Molecular Genetics, University of California, Los Angeles, CA, 90095.

## Abstract

A longstanding and still-increasing threat to the effective treatment of infectious diseases is resistance to antimicrobial countermeasures. Potentially, the targeting of host proteins and pathways essential for the detrimental effects of pathogens offers an approach that may discover broad-spectrum anti-pathogen countermeasures and circumvent the effects of pathogen mutations leading to resistance. Here we report implementation of a strategy for discovering broad-spectrum host-oriented therapies against multiple pathogenic agents by multiplex screening of drugs for protection against the detrimental effects of multiple pathogens, identification of host cell pathways inhibited by the drug, and screening for effects of the agent on other pathogens exploiting the same pathway. We show that a clinically used antimalarial drug, Amodiaquine, discovered by this strategy, protects host cells against infection by multiple toxins and viruses by inhibiting host cathepsin B. Our results reveal the practicality of discovering broadly acting anti-pathogen countermeasures that target host proteins exploited by pathogens.

Whereas medical treatments generally target specific cellular functions of patients to cure or mitigate the effects of diseases, the strategy underlying treatment of infectious disease treatment is to target the infecting pathogen^1^. Inevitably, and not surprisingly, the targeting of pathogens has led to the emergence and spread among pathogens of mutational resistance to countermeasures. Such resistance, together with a desire to expand the utility of countermeasures by increasing their range of therapeutic efficacy, has in recent years sparked interest in agents aimed at host functions that pathogens exploit to enter or be released from host cells^1^. Not infrequently, multiple pathogens or toxins that affect hosts by different mechanisms use the same host pathways^2^, raising the prospect that multiplex strategies that concurrently or sequentially screen for host functions exploited by multiple pathogenic agents may lead to the discovery of broadly active and host-oriented infectious disease countermeasures.

Here we report the discovery, using a cell-based multiplex approach to screen a library of FDA-approved drugs for the ability to interfere with disparately acting pathogens. We report here, that a compound used clinically as an antimalarial agent, inhibits both the detrimental effects of multiple bacterial toxins and the entry of Ebola and other viruses into host cells. We further show that the broad antipathogenic actions of Amodiaquine result from its ability to interfere with the functioning of the host protein, cathepsin B.

## Results

### Screening of FDA approved drugs for inhibitors of toxin - induced cell death

**Figure 1:**
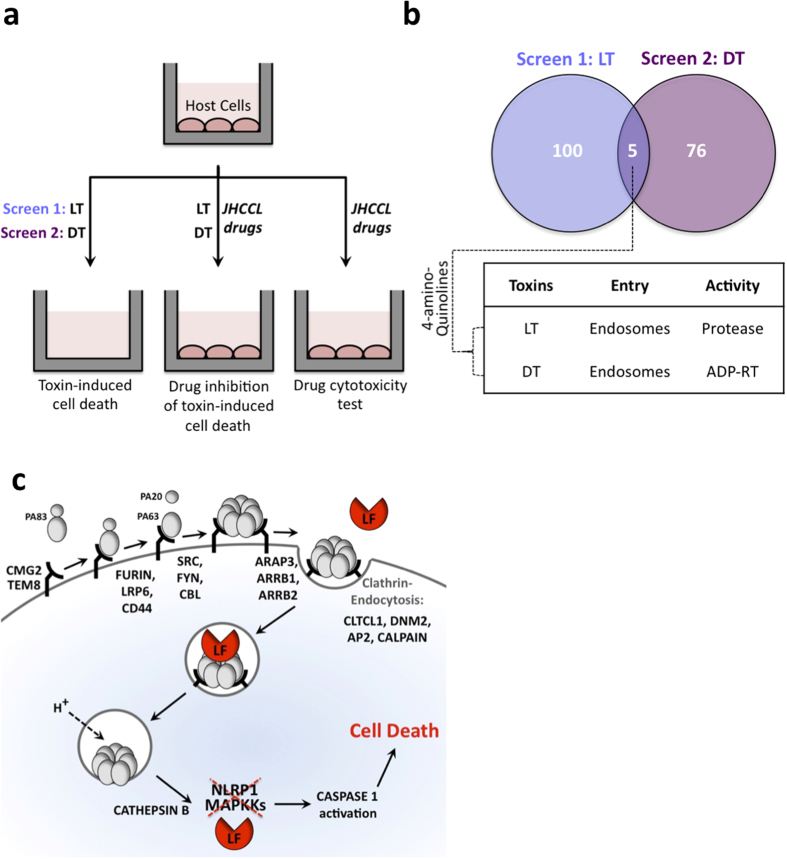
The use of Johns Hopkins Clinical Compound Library (JHCCL) to screen for inhibitors of bacterial toxins. (**a**) Schematic diagram of cellular screens to identify drugs that reduce cellular lethality induced by anthrax lethal toxin, LF and PA (LT) and diphtheria toxin (DT). (**b**) The distribution of inhibitors obtained in those screens, with a table showing the routes taken by toxins to enter into cellular cytoplasm (Endocytosis), as well as the enzymatic activities of toxins (Protease or ADP-rybosyltransferase (ADP-RT)). (**c**) Schematic depiction of the host pathway that mediates the delivery of anthrax toxin into cytoplasm. Lethal factor (LF) and edema factor (EF) interact with a third *B. anthracis*-generated protein, protective antigen (PA). Three host cell proteins CMG2, TEM8, and ITGB1 can serve as receptors for the bipartite PA/LF and PA/EF toxins. Fifteen additional host proteins are known to assist PA binding and/or internalization.

In a systematic effort to identify candidates for repurposing drugs as broad-spectrum, host-oriented, anti-toxin countermeasures, we screened members of the Johns Hopkins Clinical Compound Library (JHCCL)^3^ of 1,581 agents previously approved as drugs by the US Food and Drug Administration for the ability to reduce lethality of RAW264.7 and C32 cells treated either with *Bacillus anthracis* lethal toxin or diphtheria toxin (Fig. 1a). These toxins were chosen because the mechanisms underlying their pathogenicity are well understood and are disparate to each other.

Between 50 and 70 percent of cells used for these assays normally undergo cell death, as determined by MTT assay, within 6 and 24 hours of exposure to anthrax lethal toxin and diphtheria toxin respectively, under the experimental conditions employed. A “hit” in our screen was defined as an event where cells exposed to a compound at a concentration of 16 μM increased cell survival by at least 16 standard deviations (~1% hit rate) above the survival of control cells treated with either toxin, but is not cytotoxic to cells in the absence of toxins. Events defined as “multiplex hits” interfered with cell killing by both of the toxins (Fig. 1b). Five multiplex hits were identified and were tested further.

Anthrax toxin and diphtheria toxin enter the cytoplasm from acidified endosomes^4^. Whereas diphtheria toxin is an ADP ribosyl transferase, anthrax toxin is a protease that cleaves host MAPKK (Fig. 1c). To identify agents that inactivate host proteins exploited by toxins, we focused on hits that inhibit the mechanistically differently acting anthrax and diphtheria toxins. In order to elucidate host-targets inhibited by these drugs, we used only anthrax toxin, as the host cellular pathway that delivers the anthrax toxin into the cytoplasm is one of the best understood pathways^5^ (and Fig. 1c). Anthrax lethal toxin is an exotoxin protein complex consisting of protective antigen (PA) and lethal factor (LF), which act collectively to damage the host cell^6^. PA is an 83 kDa cellular receptor-binding protein (PA83), and the combination of PA with LF is cytotoxic^6^. LF is a 91 kDa zinc metalloprotease that cleaves the N-terminal substrate docking site of the mitogen-activated protein kinase kinases (MAP2K), preventing the passage of signals in the ERK1/2, p38, and c-Jun N-terminal kinase pathways^7^. Intoxication of a cell begins when PA83 binds to host cellular receptors, capillary morphogenesis protein 2 (CMG2), tumor endothelial marker 8 (TEM8), or integrin beta 1 (ITGB1)^2^,^8^,^9^. Once bound, host furin cleaves a 20 kDa fragment from the N-terminus of PA83, thus activating the 63 kDa protein, PA63^10^. Following activation, PA63 forms a heptamer and binds LF^11^. The toxin undergoes clathrin-mediated endocytosis and a decrease in endosomal pH induces the formation of an endosomal membrane PA channel, by which LF translocates into the cytosol^12^ before PA pores are transported to lysosomes for rapid degradation^8^,^13^,^14^. PA has been shown to induce the process of autophagy, whereby autophagosomes encapsulate endosomes and facilitate the delivery of LF into the cytoplasm^15^. A lysosomal protein, cathepsin B, is necessary for the autophagic flux, whereby LF is delivered from the intralumenal vesicles of the autophagosome-encapsulated multivesicular late endosomes into the cytoplasm through a back fusion process^14^,^16^,^17^.

**Figure 2:**
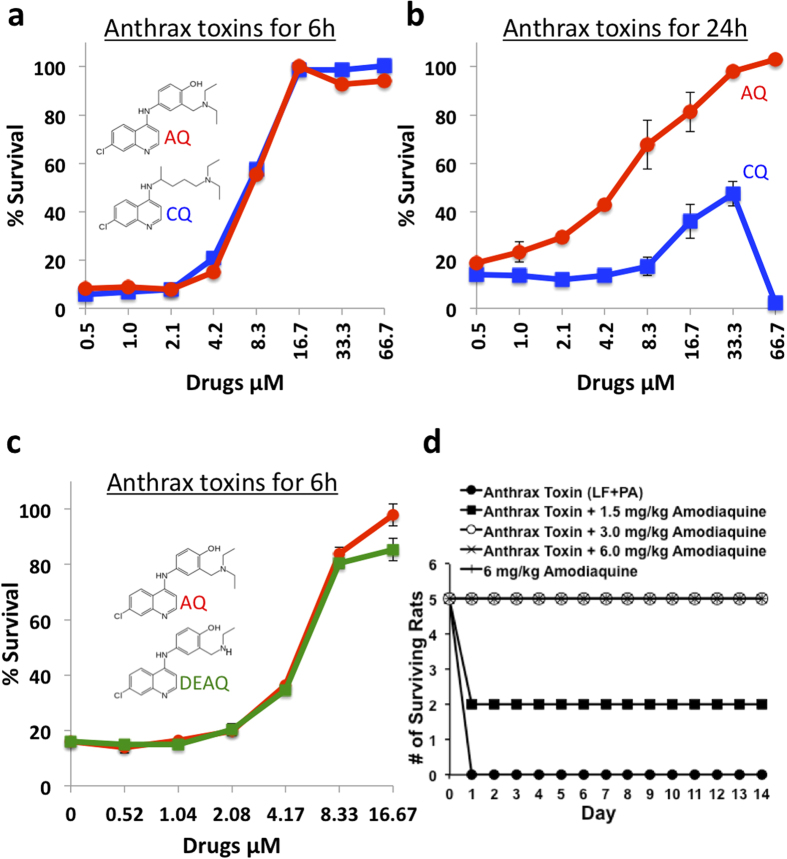
The anti-LF-PA efficacy of Amodiaquine (AQ) and its metabolite Desethylamodiaquine (DEAQ) *in intro* and *in vivo*. Amodiaquine and JHCCL available structural analogs were tested for their ability to inhibit LF-PA-mediated cytotoxicity. RAW264.7 cells were seeded at 1 × 10^4^ cells/well on 96-well plates and the following day were incubated with indicated doses of Amodiaquine or Chloroquine for 1 h, followed by either 6 h (**a**) or 24 h (**b**) intoxication with 500 ng/mL PA + 500 ng/mL LF. Cell viability was determined by MTT assay (Materials and Methods) and is shown as the percentage of survivors relative to cells not treated with drugs. AQ and DEAQ were tested for their ability to inhibit LF-PA-mediated toxicity in (**c**) RAW264.7 cells and in (**d**) Sprague-Dawley rats. RAW264.7 cells were seeded at 1 × 10^4^ cells/well on 96-well plates and the following day were incubated with indicated doses of AQ or DEAQ for 1 h, followed by 6 h intoxication with 500 ng/mL PA + 500 ng/mL LF. Survival curves of groups of male Sprague-Dawley rats challenged intravenously with 12 μg LF and 40 μg PA along with varying amounts of AQ (1.5, 3.0, and 6.0 mg/kg). Control groups of rats either received toxin only or 6.0 mg/kg of AQ only.

We observed that out of five compounds that inhibited anthrax and diphtheria toxins, two compounds were structurally related 4-amino-quinolines, Chloroquine and Amodiaquine. Two anti-malarial drugs, Chloroquine (CQ) and Amodiaquine (AQ), were observed to completely protect host cells against anthrax toxin killing in 6-hours toxin killing assay (Fig. 2a). However, our assay showed that unlike AQ, CQ only has a moderate ability to protect cells against LF-PA mediated 24-hours killing (Fig. 2b). Interestingly, none of the other quinoline-containing antimalarial compounds, Cinchonine, Primaquine, and Quinidine, from JHCCL were active (Supplementary Fig. 1a–c).

### Amodiaquine and its metabolite are potent inhibitors of LF-PA induced death *in vitro* and *in vivo*

After oral administration, AQ is rapidly absorbed and undergoes fast and extensive metabolisation by hepatocytes’ cytochrome p450 enzyme to the AQ metabolite, Desethyl-Amodiaquine (DEAQ), the main active metabolite of AQ^16^. We tested the ability of DEAQ to reduce anthrax toxin mediated cellular killing, and we observed that just like AQ, DEAQ was able to reduce toxin-mediated cytotoxicity with an EC50 of 5 μM (Fig. 2c). Since AQ and its metabolite, DEAQ, protected host cells against anthrax toxin killing, we evaluated the efficacy of AQ as a therapeutic agent during anthrax toxin intoxication in Sprague-Dawley rats. Animals were injected intravenously with a lethal dose of anthrax toxin (LD100) and were intravenously co-injected with AQ at 1.5, 3.0, or 6.0 mg/kg. The AQ doses were selected based on the FDA approved AQ dose of 10 mg/kg^18^. Animals that received a lethal dose of anthrax toxin without AQ all died within 90 minutes post intoxication (Fig. 2d). While the administration of AQ at 1.5 mg/kg saved 40% of the rats, all of the animals in that group displayed classical signs of having undergone a toxin challenge, such as ataxia, lethargy, hyperpnea, and tachypnea. Rats that were challenged with anthrax toxin and treated with AQ at 3.0 and 6.0 mg/kg all survived without displaying toxin-associated symptoms (Fig. 2d).

### Amodiaquine Inhibits Cytosolic Entry of LF

**Figure 3:**
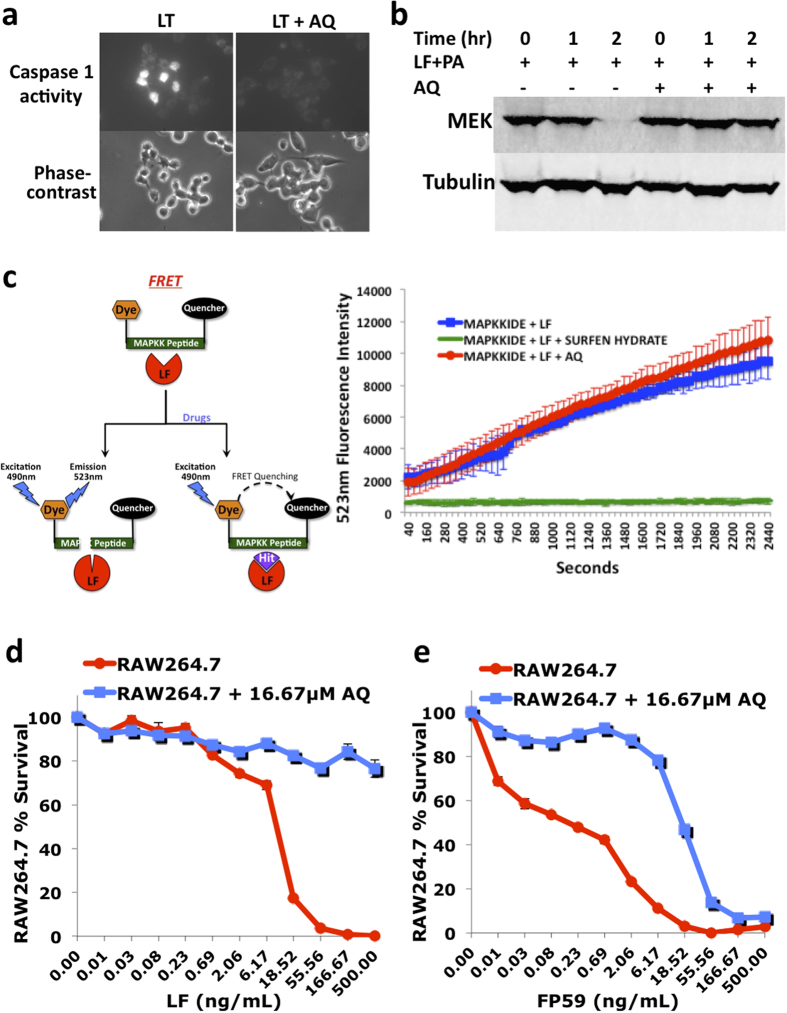
Amodiaquine inhibits cellular entry of LF. (**a**) AQ inhibits LT-mediated caspase-1 activation. RAW264.7 cells were seeded and allowed to adhere overnight, and then incubated with 16 μM AQ or DMSO for 1 h before addition of 500 ng/mL PA + LF for 2 h. FLICA was added for a further 1 h and then cells analyzed by fluorescence microscopy. (**b**) AQ inhibits LT-mediated cleavage of MEK-2. RAW264.7 cells were incubated with AQ or DMSO for 1 h before addition of vehicle control or 1 μg/mL PA + LF for up to 2 h. Cells were lysed and analyzed via immunoblotting with a MEK-2–specific antibody. Tubulin was used as a loading control. (**c**) FRET data showing fluorescence emission from three reactions, where 5.8 μg/ml LF cleaves fluorescently labeled MAPKK peptide without drugs, in the presence of a known LF inhibitor, 16 μM surfen hydrate, or in the presence of 16 μM AQ. (**d** and **e**) AQ protects cells from PA + FP59. RAW264.7 cells were preincubated with a titration of AQ for 1 h, followed by a 6 h intoxication with 500 ng/mL PA + LF (**d**) or FP59 (**e**). Cell viability was measured via MTT.

In order to identify the step at which AQ inhibits LF-PA-mediated lethality, we assessed the processes that mediate the cellular entry of this toxin and toxin-induced pyroptosis in the presence and in the absence of this drug. Caspase-1 activation, which occurs late in LF-PA intoxication, was monitored using a fluorescent probe, FLICA. This probe specifically binds to active caspase-1. While we observed high levels of caspase-1 activity upon LF-PA treatment in the absence of AQ, active caspase-1 was not detected in AQ-treated cells that were challenged with anthrax toxin (Fig. 3a). This result shows that AQ inhibits cytotoxicity upstream of caspase-1 activation.

Activation of caspase-1 by LF is a late step in pyroptosis and depends on LF catalytic activity^19^. To determine whether AQ blocks proteolysis of cellular MAPKKs by LF, we assessed the cleavage of MEK2 by immunobloting. While MEK2 was cleaved in LF-PA treated RAW264.7 cells, treatment with AQ completely prevented this effect (Fig. 3b). These results show that AQ inhibits either cytotoxicity upstream of MAPKK cleavage or blocks LF directly.

In order to test whether AQ inhibits the enzymatic activity of LF, we used a Fluorescence Resonance Energy Transfer (FRET) based assay, in which an MEK2 peptide containing a cleavage site for LF with a fluorogenic DABCYL group at the N-terminus and FITC quenching group at the C-terminus was used as LF substrate for *in vitro* assays. After cleavage by LF, the fluorescence emitted by the DABCYL increased (Fig. 3c). We tested the ability of AQ to inhibit the proteolytic activity of LF at 16 μM using FRET. In the presence of a known small molecule inhibitor of LF, surfen hydrate^20^, no emission at 523 nm was seen (Fig. 3c). In the absence of any chemical inhibitor, LF cleaved MAPKK peptide, and the fluorescence emission was observed (Fig. 3c). A similar emission at 523 nm was observed when LF was able to cleave MAPKK peptide in the presence of AQ, which shows that AQ does not block the proteolytic activity of LF at 16 μM.

AQ-treated cells were found to be less sensitive to treatment with PA + FP59 (Fig. 3d,e). FP59 is a hybrid toxin, which contains the PA binding site of LF as well as a toxin domain derived from *P. aeruginosa* exotoxin A^21^, that has been widely used as an LF surrogate, and kills cells by a different mechanism^2^. This result, along with the caspase-1 and MEK2 data, strongly suggest that AQ interferes with PA mediated toxin entry.

### Amodiaquine binds to and inhibits host cathepsin B

**Figure 4:**
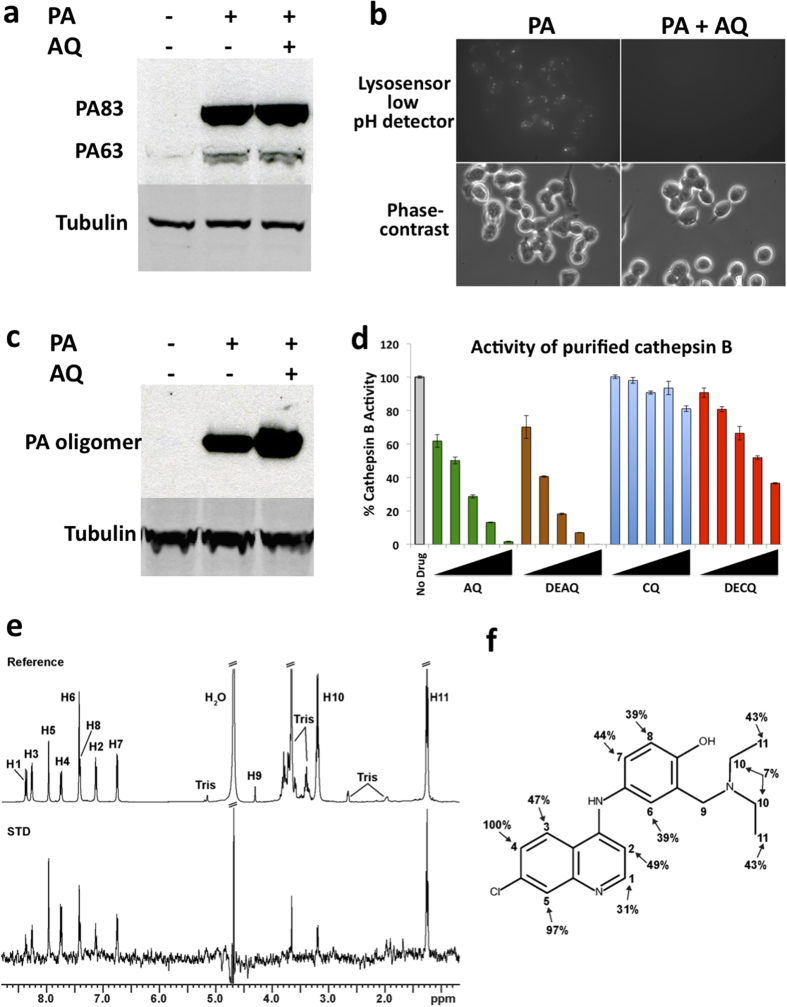
Amodiaquine inhibits host cathepsin B. (**a**) AQ does not inhibit PA binding to RAW264.7 cells. Cells were incubated with AQ for 1 h at 4 °C before addition of 1 μg/mL PA for an additional 1 h. Cells were lysed and analyzed by immunoblotting with a PA-specific antibody. (**b**) AQ neutralizes acidic vesicles. RAW264.7 cells were pre-treated with AQ or vehicle control for 1 h and then treated with 500 ng/ml of PA for an additional hour at 37 °C before addition of Lysosensor Green DND-189 for a further 10 min. Cells were then visualized by fluorescence microscopy. (**c**) AQ results in increased abundance of PA pores. Cells were pre-treated with AQ for 1 h at 37 °C and then were exposed to 1 μg/mL of PA at 37 °C for 1 h. Cells were lysed and analyzed by immunoblotting with a PA-specific antibody. (**d**). FRET assay showing the activity of purified human cathepsin B without drugs, or with addition of AQ, DEAQ, CQ, or DECQ at 4, 8, 16, 33, or 66 μM. (**e**) ^1^H-NMR spectra of AQ in the presence of cathepsin B. The reference spectrum of AQ with its atoms labeled (top), and the STD spectrum (bottom) are shown. The data were collected using a protein:ligand ratio of 1:100 with on- and off-resonance saturation at 0.04 ppm and 30 ppm, respectively. (**f**) Chemical structure of AQ showing the atom-specific magnitude of the STD effects. The STD effect was calculated according to the formula *A*_*STD*_* *=* *(*I*_*0*_ − *I*_*sat*_)/*I*_*0*_. All STD effects are expressed as a percentage relative to the H4 atom (100%).

In order for LF to reach the cytosol, PA83 must bind to host cell receptors, be cleaved by furin into PA63, heptamerize into a pre-pore form, bind LF, and proceed to low-pH endosomes where PA-heptamers undergo an acid-dependent conformational change from pre-pore to pore^22^. Host-cell binding of PA and its proteolytic cleavage were monitored by immunoblot in the presence and in the absence of AQ. We observed that AQ did not block binding of PA83 to cells or block proteolytic processing of PA to generate PA63 (Fig. 4a). The inability of AQ to block the activity of host furin cleavage of PA83 is consistent with our observation that AQ equally reduces sensitivity of host cells to both PA83 + LF and PA63 + LF treatments (Supplementary Fig. 2). These results indicate that AQ blocks intoxication at a step downstream of PA binding and assembly on the host-cell surface.

CQ was previously shown to prevent endosomal acidification^23^. Since AQ is structurally similar to CQ, we tested whether AQ neutralizes anthrax toxin induced endosomal acidification. We used Lysosensor Green DND-189 to probe acidic organelles in the cells. Lysosensor is a green fluorescent dye used for tracking acidic organelles in the cell. Neutralization of these compartments can be visualized as a loss in punctate-fluorescent structures. In the absence of AQ, PA-treated cells displayed Lysosensor fluorescence, and AQ markedly decreased cell-associated Lysosensor fluorescence (Fig. 4b). These results predict that AQ may block the ability of PA to either access acidified endosomes or to form PA pores. It is known that the pore formation of PA, which results from exposure to acidic pH within endosomes, is resistant to dissociation by SDS and runs as an oligomer on SDS-PAGE^22^. Surprisingly, treatment of cells with AQ resulted in higher, rather than lower, abundance of SDS-resistant PA-oligomers compared to cells treated with PA only (Fig. 4c).

A similar observation was reported by Ha *et al.*^14^, who showed that an inhibition of host lysosomal cathepsin B by an unrelated small molecule CA-074, resulted in (i) elevated accumulation of PA pores in late endosomes, (ii) the inability of LF to be released from the late endosomes into the cytoplasm, and (iii) results in reduction of cellular sensitivity to LF-PA. Upon formation of the SDS-resistant PA63 pore in acidic endosomes, PA pores are then transported to lysosomes for rapid degradation^24^. Ha *et al.* showed that cathepsin B mediates the fusion of lysosomes with endosomes, and that this fusion is necessary for the release of LF from the endosomes into the cytoplasm^14^. In order to gain further insight into the mechanism of AQ-mediated protection of cells against anthrax toxin killing, we tested whether AQ inhibits cathepsin B protease activity of purified human cathepsin B using a FRET assay. We observed that both AQ and DEAQ directly inhibit cathepsin B activity in a dose dependent manner without drug pre-incubations, at drug concentrations used in cellular experiments (Fig. 4d). We tested whether AQ inhibits cathepsin B in RAW264.7 cells. We observed that cells pre-treated with AQ or with DEAQ for 1 hour lost cathepsin B enzymatic activity in a dose dependent manner (Supplementary Fig. 3a). In addition, we tested the ability to AQ to inhibit cathepsin B activity in a protein lysate from cells that were not exposed to drugs prior to the lysis, and we observed that AQ and DEAQ inhibited cathepsin B activity (Supplementary Fig. 3b). We determined that the addition of drugs to cathepsin B reactions did not change the pH (pH 5.8) of the reaction buffer. In all of the cathepsin B experiments we observed that CQ and its metabolite, Desethyl-Chloroquine (DECQ), were weaker inhibitors of cathepsin B activity (Fig. 4d and Figure S3), which corresponds to the phenotypic data seen in Fig. 2.

The interaction between AQ and cathepsin B was probed using saturation transfer difference NMR spectroscopy (STD). This technique is a powerful label-free ligand-observed tool to study molecular interactions in solution^25^,^26^. STD relies on the transfer of selective saturation (excitation) of the protein’s atoms to atoms located in the bound ligand. Two 1D ^1^H-NMR spectra are acquired—one in which the protein’s signals are selectively saturated (“on” resonance, I_sat_), and another in which no signals are saturated (“off” resonance, I_0_). Ligand binding is evident by the difference spectrum (I_0_ − I_sat_), as only when binding occurs are the signals of the ligand affected. Figure 4e shows the ^1^H-NMR spectrum of 2 mM AQ in the presence of 20 μM cathepsin B (ligand:protein molar ratio of 100:1), and its corresponding STD difference spectrum. The presence of AQ signals in the difference spectrum clearly indicates that it is binding to the enzyme. An inspection of the atom-specific effects provides insight into the mode of binding. Atoms in the quinoline backbone display the largest reduction in their intensities, suggesting that they are in close proximity to cathepsin B (Fig. 4f, atoms H4 and H5 exhibit STD effects of 100 and 97%, respectively). In contrast, the H9 and H10 methylene protons are modestly affected, suggesting that in the complex they are located distal to hydrogen atoms in the protein. Interestingly, several atoms in the phenol ring display substantial STD effects suggesting that they are near the protein. This is consistent with the lower biological activities of AQ analogs that remove the phenol ring (Fig. 2 and Supplementary Fig. 1). The importance of the AQ quinoline ring revealed by STD is also compatible with the crystal structure of cathepsin B bound to nitroxoline, as this small molecule contains a quinoline moiety that interacts extensively with the enzyme^27^.

### Amodiaquine inhibits pathogenicity of Ebola virus

Upon observing that AQ protects cells from anthrax and diphtheria toxins, we hypothesized that AQ and DEAQ might also be able to inhibit the entry of Ebola virus. Ebola virus, among other viruses, requires low endosomal pH^28^ as well as host cathepsin B function^29^ for membrane fusion and infection of host cells. In fact, AQ and CQ have recently been reported to inhibit Ebola virus pathogenicity in cells^28^,^29^. We re-tested AQ and CQ’s ability to inhibit Ebola virus abundance in infected HeLa cells by visualizing infected cells using immune-staining, and in addition we tested the ability of their metabolites, DEAQ and DECQ, to inhibit Eboa virus propagation in cultured cells *in vitro*. To test AQ-mediated inhibition of viral propagation, we compared infection by Ebola virus (Kikwit) at MOI of 0.5 with and without drugs for 48 hours. To detect infected cells, immuno-staining was completed with anti-Ebola-glycoprotein antibodies. AQ and DEAQ effectively inhibited propagation of Ebola virus in HeLa cells with EC50’s in the low μM range (Table 1): the EC50’s of AQ and DEAQ were 3.8 and 3.6 μM respectively. The EC50’s of CQ and DECQ in HeLa were 4.8 and 7.3 μM respectively. This data shows that DEAQ is more efficacious in inhibiting Ebola propagation compared to DECQ. Similar results were obtained by testing AQ, CQ, DEAQ, and DECQ for their ability to inhibit Ebola virus in primary human cell line, HFF-1 (Supplementary Table 1). This result confirms that anti-viral effect of AQ is not related to its effects on the host cell cycle or cellular proliferation.

The life cycle of Ebola virus in cultured host cells is 20–24 hours. To more clearly evaluate effects of AQ and DEAQ on a single viral life cycle, we compared the inhibitory effects of these drugs after 24 and 48 hours of Ebola virus infection in cells. We observed that AQ and DEAQ are at least two times more potent after 24 h of infection (EC50’s 2 μM), compared to 48 h, during which time the secondary round of infection occurs and possibly slightly decreases the antiviral efficacies of AQ and DEAQ (EC50’s 4 μM) (Table 2).

### Amodiaquine inhibits pathogenicity of other Category A, B, and C pathogenic agents that enter into host cytoplasm from acidified endosomes

In addition to anthrax toxin^15^, diphtheria toxin^30^, and Ebola virus^29^,^31^, other pathogenic agents SARS coronavirus^31^, Venezuelan equine encephalitis virus (VEEV)^32^, Rabies virus^33^, Junin virus^34^, Chikungunya virus^35^, and *Clostridium difficile* toxin B^36^ enter the cytoplasm from endosomes and all require the acidification of endosomes. We demonstrated that all of those pathogenic agents are inhibited by AQ in their respective *in vitro* cellular assays (Table 3 and Supplementary Fig. 4a). We also tested the ability of DEAQ to inhibit pathogenicity of Junin and Chikungunya viruses, and observed that DEAQ inhibits those two viruses with EC50’s similar to those of AQ (Supplementary Table 2).

Other pathogenic agents, including cholera toxin^37^, *Pseudomonas aeruginosa* exotoxin A^38^, Poliovirus 3^18^, and Herpes simplex virus 1^39^ are transported in a retrograde fashion to the endoplasmic reticulum (ER) and retrotranslocated into the cytoplasm by the host ER-associated degradation pathway^37^,^38^. We observed that cytotoxicity mediated by those pathogens was not blocked by AQ (Table 3 and Supplementary Fig. 4b,c). In addition, the inability of AQ to inhibit cholera and *Pseudomonas* toxins, which are both ADP-rybosyltransferases, suggests that AQ’s adverse effect on diphtheria toxin (Fig. 1), which is also ADP-rybosyltransferase, occurs by inhibition of host cathepsin B only.

Another group of pathogens enter the cytoplasm by ways that do not rely on the acidification of endosomes, and include Human Cytomegalovirus^40^ and Respiratory syncytial virus^41^. We determined that AQ did not reduce the pathogenicity of those viruses (Table 3). This data supports the conclusion that AQ inhibits the entry of toxins into the cytoplasm from acidified endosomes.

## Discussion

**Figure 5:**
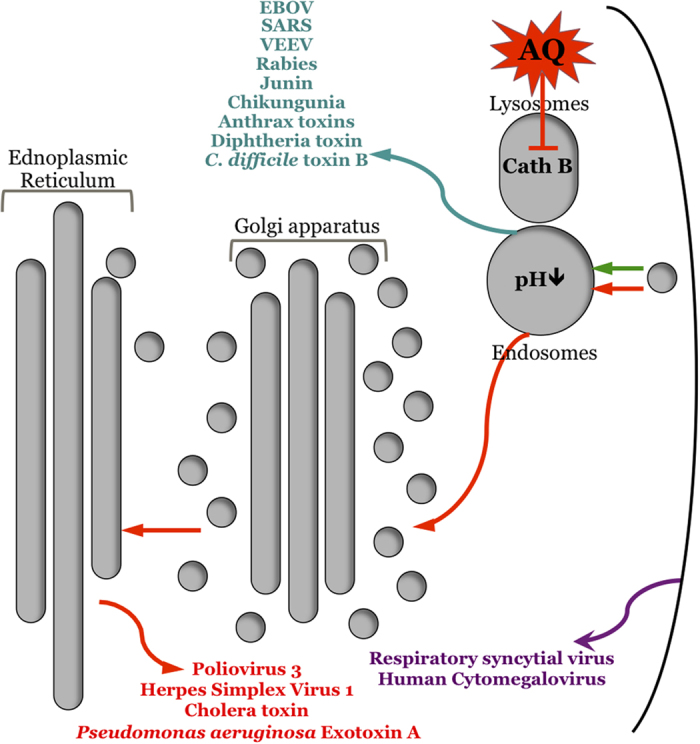
Mechanism of action of Amodiaquine. AQ reduces the activities of pathogens that enter into host cytoplasm from acidified endosomes by exploiting cellular cathepsin B. AQ does not inhibit the entry of pathogens that enter through a retrograde fashion to the endoplasmic reticulum (ER) and retrotranslocated into the cytoplasm by the host ER-associated degradation pathway or by pH-independent entry into cytoplasm.

The discovery and development of novel drugs against biological threat agents such as *Bacillus anthracis* and Ebola virus, is an important concern worldwide. We have shown that AQ is a broad-spectrum drug that could be applied rapidly to cure biological emergencies caused by the several identified pathogens (Fig. 5). For example, the current Ebola crisis has already killed more than 10,000 people in 2014 and 2015. Repurposing of a compound with well-established safety and pharmacokinetic profiles—as well as proven high-volume GMP manufacturing—may be the only way that a therapy can be tested and deployed in time to protect the 21 + million people in the three hardest-hit West African countries.

To help address this Ebola crisis, as we were preparing this manuscript, we communicated our discoveries to Médecins Sans Frontières (MSF, Doctors Without Borders), and encouraged them to explore whether the administration of AQ in West African countries hit by Ebola epidemic is associated with a reduced mortality due to Ebola infections. MSF conducted a study and observed that the administration of AQ provided a substantial protection against Ebola mortality and reduced the mortality risk by 31%, and in this study MSF acknowledged the authors of this study (https://www.youtube.com/watch?v=rTyoc4s1Mco).

As seen in current Ebola epidemic, the human host can be co-exposed to numerous deadly pathogens and thus the infected individual will need to take numerous countermeasures (each with their own side-effects) to protect against multiple infectious organisms. Therefore, therapies capable of inhibiting multiple pathogens are needed. Broad-spectrum anti-pathogen drugs, capable of simultaneously inhibiting numerous unrelated pathogens, could be developed by targeting host proteins commonly exploited by those pathogens. In this work we show that AQ inhibits the host target rather than the pathogen, which makes AQ host-oriented broad-spectrum antimicrobial that is less likely to be circumvented by pathogen mutations that lead to microbial resistance to the countermeasure.

AQ was discovered by Parke-Davis in the 1940s and was approved by the FDA as an anti-malarial in the late 1940s and early 1950s under the trade name Camoquine. AQ is currently on the WHO’s Model List of Essential Medicines. Today AQ is recommended as a frontline anti-malarial therapy in combination with artemisinin. AQ belongs to the “quinoline antimalarials” (Reviewed in^42^). AQ inhibits *Plasmodium falciparum* phosphoethanolamine methyltransferase, an enzyme found in malarial parasites but not in humans^43^. Here we report that AQ also binds and blocks the function of host cathepsin B protein and thus entry of key pathogens into the cytoplasm.

Since AQ has been used for the past 60 years, its safety and pharmacokinetic profiles are well known. For example, it has been determined that after oral administration in healthy subjects, AQ is quickly absorbed and biotransformed into its main active form, DEAQ, which in turn is slowly eliminated with a terminal half-life of 9–18 days. The availability of PK values for AQ allows us to compare it to our *in vitro* data. The EC50 values for all of the tested pathogens inhibited by AQ or by DEAQ were at low μM range. Importantly, the known Cmax values (maximal amounts of drug in the blood after oral administration) of DEAQ in humans are in low μM range^43^,^44^,^45^ and are comparable to the DEAQ EC50 values found *in vitro* for tested pathogens. This suggests that it is possible to achieve anti-pathogen effective AQ doses in blood by orally administered AQ.

## Methods

### Chemicals and Reagents

All toxins were purchased from List Biological Laboratories. FP59 was a kind gift from S. Leppla (National Institute of Allergy and Infectious Diseases/National Institutes of Health, Bethesda, MD). An FDA-approved drug library comprising of 1,581 drugs was purchased from Johns Hopkins, titled, Johns Hopkins Clinical Compound Library (JHCCL) version 1.0. The drugs arrived as 10 mM stock solutions in sealed microtiter plates and were made using DMSO or water as solvents. Drugs were arrayed in 96-well plates and screened at a stock concentration of 10 mM. The library was stored at −20 °C until use. Prior to use, the library of drugs was thawed at 25 °C. Compounds that were determined to be compounds of interest, were isolated and reproduced from 10 mM solutions. Amodiaquine, Chloroquine, Cinchonine, Primaquine, and Quinidine were purchased from Sigma-Aldrich (St. Louis, MO, USA). All drugs were prepared at 10 mM using DMSO as a solvent. Desethyl-amodiaquine and desethyl-chloroquine were purchased from Toronto Research Chemicals Inc. Anti–N-terminal MEK-2, anti-tubulin, and anti-PA antibodies were purchased from Santa Cruz Biotechnology. Purified human cathepsin B protein used for NMR experiments was purchased from ACROBiosystems.

### Cellular Drug Screens

RAW264.7-pGIPZ(−)^2^ mouse macrophage cells were maintained in DMEM (Sigma-Aldrich) supplemented with 10% FBS (Bioexpress) and 100 μg/mL penicillin and 100 μg/mL streptomycin. RAW264.7-pGIPZ(−) cells (10,000 per well) were seeded in 96-well plates (100 μl/well) 24 hours before the assay. During the drug library screen assay, 0.75 μl of 3.3 mM drugs were added to 150 μl of cell-containing media to achieve 16 μM of each compound per well. Cells were treated with compounds for 1 hour at 37 °C 5%CO_2_, and then challenged with anthrax toxins, such that the final toxins concentrations were 0.5 μg/ml. Cells were treated with toxin and drugs for 6 hours. As rodent cells are resistant to diphtheria toxin, C32 human melanoma cell line was used for diphtheria toxin screening, where cells were treated with 2 μg/ml for 24 hours. Determination of cell viability by 3-(4,5-dimethylthiazol-2-yl)-2,5-diphenyltetrazolium bromide (MTT) assay was performed as described^46^. Cell viability is shown as the percentage of surviving cells obtained relative to cells treated with DMSO (100%).

### FRET MAPKKide LF activity

For drug testing in 96-well plates, the reaction volume was 250 μl per well, containing 20 mM HEPES pH 7.2, 5 μM MAPKKide conjugated with DABYL and FITC (List Biological Laboratories, Inc), and 3.3 μM of JHCCL compound. The reaction was initiated by adding LF to a final concentration of 6 μg/ml. Kinetic measurements were obtained at 37 ºC every 40 sec for 40 min using a fluorescent plate reader. Excitation and emission wavelengths were 490 nm and 523 nm, respectively, with a cutoff wavelength of 495 nm.

### Toxins Treatments

Cells were seeded in a 96-well plate at a density of 10,000 cells/well 1 d before toxin treatment. Various concentrations of LF or FP59 combined with a fixed concentration of PA (500 ng/mL) were added to the wells, and cells were incubated for 6 h at 37 °C. Cell viability was measured by MTT assay. In other toxin treatment experiments, cells were pre-treated with various concentrations of drugs, and then treated with constant concentrations of 8.3 μg/ml of *C. difficile* toxin B, 500 ng/ml of *P. aeruginosa* Exotoxin A, or 500 ng/ml of Cholera toxin for 6 hours. RAW264.7 survival was measured by MTT assay. Each data point shown for MTT assays indicates the mean ± SD value obtained in triplicate assays done in a representative experiment. At least three such experiments were routinely carried out.

### MEK Cleavage Assay

RAW264.7 cells were pre-treated with 66.67 mM of AQ for 1 hour. Following the pre-treatment, the cells exposed to 1 mg/mL of PA and LF at 37 °C for up to two hours in the presence of 16.67 μM of AQ. The cells were then washed with cold PBS for 5× and lysed with RIPA buffer containing a protease inhibitor mixture (Roche). Cell lysates were quantified using the BCA protein quantification kit (Pierce) and loaded onto 4–12% denaturing gels (Criterion XT Precast Gel, Bio-Rad). After electrophoresis for several hours, the gel was transferred overnight to nitrocellulose membranes; membranes were probed with anti-MEK-2 or anti tubulin antibodies. Quantitative Western blot analyses of the bands were accomplished using the VersaDoc 1000 instrument (Bio-Rad) or Odyssey infrared imaging system (LI-COR Biosciences).

### Biochemical Assay of PA Binding and Internalization

Cells were pre-treated with AQ as described above for 1 h, either at 4 °C for PA binding or at 37 °C for PA internalization assays cells were exposed to 1 μg/mL of PA at 4 °C for 1 h for binding assay or at 37 °C for 1 h for internalization assay. Cells were then washed with PBS solution three times and lysed in RIPA buffer containing a protease inhibitor mixture (Roche). Western blot analysis was performed using anti-PA antibody anti-tubulin monoclonal antibody (Sigma-Aldrich). Chemiluminescence of bands and their relative intensities were revealed using a VersaDoc 1000 instrument (Bio-Rad).

### Caspase-1 Activity Assay

Cells were treated with AQ for 1 h before addition of 500 ng/ml LF-PA for an additional 2 h. FAM FLICA™ Caspase 1 Assay Kit was obtained from ImmunoChemistry Technologies LLC. FLICA reagent was added for 1 h. FLICA fluorescence was visualized as follows: cells were washed three times in PBS, fixed in 4% (wt/vol) paraformaldehyde, and examined under a fluorescence microscope (DM5500 B; Leica).

### Staining Cells with Lysosensor Green DND-189

Cells were treated with AQ for 1 h before an addition of 500 ng/ml PA for an additional hour and then stained with Lysosensor Green DND-189 for 10 min at 37 °C. Cells were then washed and fluorescence was visualized as follows: cells were fixed in 4% (wt/vol) paraformaldehyde, and examined under a fluorescence microscope (DM5500 B; Leica).

### Cathepsin B Activity Assay

Cathepsin B activity in total cell lysates was determined using an InnoZyme ™ cathepsin B activity assay kit (EMD Milipore) and performed according to the manufacturer’s instruction. Cathepsin B activity in cellular lysates was tested in the following way: RAW264.7 cells untreated with drugs were lysed, and equal amount of cathepsin B containing protein lysate was added to the substrate solution (EMD Milipore) with and without AQ, CQ, DEAQ, or DECQ at concentrations of 4, 8, 16, 33, or 66 μM. Cellular cathepsin B activity with and without drugs was tested by pre-treating cells with drugs for 1 hour, followed by lysing cells and testing cathepsin B activity with a fluorescently labeled substrate. The activity of 0.5 ng/μl of purified human cathepsin B was mixed with and without drugs without pre-incubation and detected with a fluorescently labeled substrate. Fluorescence intensity indicating cathepsin B activity was measured at an excitation wavelength of 370 nm and emission wavelength of 450 nm (Molecular Devices, Spectra Max 384 PLUS).

### Rat intoxication challenge

The rat studies were performed at Explora Biolabs, San Diego, CA following Institutional Animal Care and Use Committee (IACUC) approved protocols. Five Male Sprague-Dawley rats (226 to 250 g; Charles River) per group were used. The toxin mixture was prepared for each group by mixing 12 μg of LF with 40 μg of PA or with 1.5, 3.0, or 6.0 mg/kg of Amodiaquine in a 500-μl volume per rat. Rats were monitored for signs of clinical illness or death for 14 days after the challenge.

### NMR spectroscop

All STD experiments were performed at 25 °C using a Bruker Avance 500-MHz spectrometer equipped with a cryogenic probe. The pulse scheme employed excitation sculpting with gradients, and a 50 ms spin lock filter (T_1ρ_) to suppress signals originating from the water and protein signals, respectively^25^. An irradiation power of 26 Hz was applied on-resonance at 0.04 ppm, and off-resonance at 30 ppm, for a total saturation time of 4 s. Spectra were collected in an interleaved manner to account for any temporal fluctuations. Separate control experiments were performed using samples of cathepsin B or Amodiaquine to confirm the selectivity of saturation. STD spectra were acquired with 4096 scans and 32 k data points using a spectral width of 7002.8 Hz centered at 2352 Hz. All experiments were performed in deuterated tris buffer: 50 mM D_11_-Tris, 150 mM NaCl, pH 7.5 supplemented with 8% D_2_O. The sample used for STD measurements contained 20 mM and 2 mM cathepsin B and Amodiaquine, respectively. The STD NMR data were processed and analyzed using Topspin 3.1 software (Bruker, Billerica Ma). STD effects were calculated according to the formula: A_STD_ = (I_0_ − I_sat_)/I_0_, where I_sat_ and I_0_ are the intensity of the Amodiaquine signal recorded with and without saturation of the protein, respectively.

### Viral tests

The procedures of all viral experiments are described in supplemental materials and methods. Specifically, infection of HeLa cells with Ebola was done using Ebola virus (Kikwit) MOI = 0.5 (calculated for 4,000 cells/well, assuming one complete round of replication of HeLa cells at 15 ± 2 hrs after cell seeding). Cells were incubated with the virus for 24 or 48 h. Infection was terminated by fixing samples in formalin solution, and immuno-staining was used to visualize infected cells. Cells were treated with anti-GP specific monoclonal antibody (6D8) (1 to 1000 dilution) followed by anti-mouse IgG conjugated with Dylight488 (Thermo) (1 to 1000 dilution) in blocking buffer containing 3% BSA in PBS. Nuclei were stained with Draq5 (Biostatus) diluted in PBS buffer. Images were acquired on the Opera imaging instrument (Perkin Elmer) using 10× Air objective and four images/well were acquired and analyzed using PE Acapella algorithms.

## Additional Information

**How to cite this article**: Zilbermintz, L. *et al.* Identification of agents effective against multiple toxins and viruses by host-oriented cell targeting. *Sci. Rep.* **5**, 13476; doi: 10.1038/srep13476 (2015).

## Supplementary Material

Supplementary Information

## Figures and Tables

**Table 1:** The effect of AQ, CQ, and its metabolites, DEAQ and DECQ, on the pathogenicity of EBOV in HeLa cells.

Pathogen	Drug, hours	EC50μM	SDμM	CC50μM	SI50
EBOV	AQ, 48 h	3.8	0.38	50	13.2
EBOV	DEAQ, 48 h	3.6	0.35	50	13.9
EBOV	CQ, 48 h	4.8	0.57	100	20.7
EBOV	DECQ, 48 h	7.3	0.55	100	13.8

The ability of drugs to reduce the abundance of Ebola virus (EBOV) in host cells was measured in cells by fluorescent microscopy.

**Table 2:** The effect of time of AQ and its metabolite treatments on the pathogenicity of EBOV.

Pathogen	Drug, hours	EC50μM	SDμM	CC50μM	SI50
EBOV	AQ, 24 h	2.38	0.47	>50	>21
EBOV	AQ, 48 h	4.46	0.44	>50	>11.2
EBOV	DEAQ, 24 h	2.15	0.48	>50	>23.3
EBOV	DEAQ, 48 h	4.12	0.36	>50	>12.1

The 50% effective (EC50, virus-inhibitory) concentrations and 50% cytotoxic (CC50, cell-inhibitory) concentrations were determined. CC50 divided by EC50 indicate the selectivity index (SI) value.

**Table 3:** Sensitivities of Category A, B, and C pathogens to Amodiaquine.

Pathogen	EC50 μM	CC50 μM	SI50
Pathogens known to enter into cytoplasm from Endosomes, low pH-dependent			
Ebola Virus	2.4	>50	>20
SARS coronavirus	2.4	32	13.3
Venezuelan equine encephalitis virus	3.2	31	9.7
Rabies	5.7	>10	>2
Junin	16.9	50	3.0
Chikungunya	18.3	>50	>2
*Clostridium difficile* Toxin B	4.6	30.7	6.7
Entry into cytoplasm from Endoplasmic Reticulum			
Poliovirus 3	>100	>100	1
Herpes simplex virus 1	>60	>60	1
Cholera Toxin	>66.7	>66.7	1
*Pseudomonas aeruginosa* Exotoxin A	>66.7	>66.7	1
Pathogens known to enter into cytoplasm, low pH-independent			
Human Cytomegalovirus	>60	>60	1
Respiratory syncytial virus	32	32	1

The ability of drugs to reduce the abundance of the indicated pathogens viruses or cytotoxicity induced by bacteria toxins in host cells. The analysis was done as in Table 1.

## References

[b1] Bekerman E. & Einav S. Infectious disease. Combating emerging viral threats. Science 348, 282–283 (2015). 25883340 10.1126/science.aaa3778 PMC4419706

[b2] Martchenko M., Jeong S. Y. & Cohen S. N. Heterodimeric integrin complexes containing beta1-integrin promote internalization and lethality of anthrax toxin. Proc Natl Acad Sci USA 107, 15583–15588 (2010). 20713715 10.1073/pnas.1010145107 PMC2932583

[b3] Chong C. R., Chen X., Shi L., Liu J. O. & Sullivan D. J. Jr. A clinical drug library screen identifies astemizole as an antimalarial agent. Nature chemical biology 2, 415–416 (2006). 16816845 10.1038/nchembio806

[b4] Sandvig K. & van Deurs B. Delivery into cells: lessons learned from plant and bacterial toxins. Gene Ther 12, 865–872 (2005). 15815697 10.1038/sj.gt.3302525

[b5] Martchenko M., Candille S. I., Tang H. & Cohen S. N. Human genetic variation altering anthrax toxin sensitivity. Proc Natl Acad Sci USA 109, 2972–2977 (2012). 22315420 10.1073/pnas.1121006109 PMC3286947

[b6] Moayeri M. & Leppla S. H. Cellular and systemic effects of anthrax lethal toxin and edema toxin. Mol Aspects Med 30, 439–455 (2009). 19638283 10.1016/j.mam.2009.07.003 PMC2784088

[b7] Duesbery N. S. *et al.* Proteolytic inactivation of MAP-kinase-kinase by anthrax lethal factor. Science 280, 734–737 (1998). 9563949 10.1126/science.280.5364.734

[b8] Bradley K. A., Mogridge J., Mourez M., Collier R. J. & Young J. A. Identification of the cellular receptor for anthrax toxin. Nature 414, 225–229 (2001). 11700562 10.1038/n35101999

[b9] Scobie H. M., Rainey G. J., Bradley K. A. & Young J. A. Human capillary morphogenesis protein 2 functions as an anthrax toxin receptor. Proc Natl Acad Sci USA 100, 5170–5174 (2003). 12700348 10.1073/pnas.0431098100 PMC154317

[b10] Klimpel K. R., Molloy S. S., Thomas G. & Leppla S. H. Anthrax toxin protective antigen is activated by a cell surface protease with the sequence specificity and catalytic properties of furin. Proc Natl Acad Sci USA 89, 10277–10281 (1992). 1438214 10.1073/pnas.89.21.10277 PMC50321

[b11] Kintzer A. F. *et al.* The protective antigen component of anthrax toxin forms functional octameric complexes. J Mol Biol 392, 614–629 (2009). 19627991 10.1016/j.jmb.2009.07.037 PMC2742380

[b12] Thoren K. L. & Krantz B. A. The unfolding story of anthrax toxin translocation. Mol Microbiol 80, 588–595 (2011). 21443527 10.1111/j.1365-2958.2011.07614.x PMC3094749

[b13] Checroun C., Wehrly T. D., Fischer E. R., Hayes S. F. & Celli J. Autophagy-mediated reentry of Francisella tularensis into the endocytic compartment after cytoplasmic replication. Proc Natl Acad Sci USA 103, 14578–14583 (2006). 16983090 10.1073/pnas.0601838103 PMC1600002

[b14] Ha S. D., Ham B., Mogridge J., Saftig P., Lin S. & Kim S. O. Cathepsin B-mediated autophagy flux facilitates the anthrax toxin receptor 2-mediated delivery of anthrax lethal factor into the cytoplasm. J Biol Chem 285, 2120–2129 (2010). 19858192 10.1074/jbc.M109.065813 PMC2804368

[b15] Tan Y. K., Kusuma C. M., St John L. J., Vu H. A., Alibek K. & Wu A. Induction of autophagy by anthrax lethal toxin. Biochem Biophys Res Commun 379, 293–297 (2009). 19103170 10.1016/j.bbrc.2008.12.048

[b16] Abrami L., Lindsay M., Parton R. G., Leppla S. H. & van der Goot F. G. Membrane insertion of anthrax protective antigen and cytoplasmic delivery of lethal factor occur at different stages of the endocytic pathway. J Cell Biol 166, 645–651 (2004). 15337774 10.1083/jcb.200312072 PMC2172425

[b17] Sobo K. *et al.* Late endosomal cholesterol accumulation leads to impaired intra-endosomal trafficking. PLoS One 2, e851 (2007). 17786222 10.1371/journal.pone.0000851 PMC1952175

[b18] Tershak D. R. Association of poliovirus proteins with the endoplasmic reticulum. Journal of virology 52, 777–783 (1984). 6092710 10.1128/jvi.52.3.777-783.1984 PMC254596

[b19] Wickliffe K. E., Leppla S. H. & Moayeri M. Anthrax lethal toxin-induced inflammasome formation and caspase-1 activation are late events dependent on ion fluxes and the proteasome. Cell Microbiol 10, 332–343 (2008). 17850338 10.1111/j.1462-5822.2007.01044.x PMC2515708

[b20] Panchal R. G. *et al.* Identification of small molecule inhibitors of anthrax lethal factor. Nat Struct Mol Biol 11, 67–72 (2004). 14718925 10.1038/nsmb711

[b21] Arora N., Klimpel K. R., Singh Y. & Leppla S. H. Fusions of anthrax toxin lethal factor to the ADP-ribosylation domain of Pseudomonas exotoxin A are potent cytotoxins which are translocated to the cytosol of mammalian cells. J Biol Chem 267, 15542–15548 (1992). 1639793

[b22] Miller C. J., Elliott J. L. & Collier R. J. Anthrax protective antigen: prepore-to-pore conversion. Biochemistry 38, 10432–10441 (1999). 10441138 10.1021/bi990792d

[b23] Squires R. C., Muehlbauer S. M. & Brojatsch J. Proteasomes control caspase-1 activation in anthrax lethal toxin-mediated cell killing. J Biol Chem 282, 34260–34267 (2007). 17878154 10.1074/jbc.M705687200

[b24] Liu S. & Leppla S. H. Cell surface tumor endothelium marker 8 cytoplasmic tail-independent anthrax toxin binding, proteolytic processing, oligomer formation, and internalization. J Biol Chem 278, 5227–5234 (2003). 12468536 10.1074/jbc.M210321200

[b25] Mayer M. & Meyer B. Group epitope mapping by saturation transfer difference NMR to identify segments of a ligand in direct contact with a protein receptor. J Am Chem Soc 123, 6108–6117 (2001). 11414845 10.1021/ja0100120

[b26] Meyer B. & Peters T. NMR spectroscopy techniques for screening and identifying ligand binding to protein receptors. Angew Chem Int Ed Engl 42, 864–890 (2003). 12596167 10.1002/anie.200390233

[b27] Mirkovic B. *et al.* Novel mechanism of cathepsin B inhibition by antibiotic nitroxoline and related compounds. ChemMedChem 6, 1351–1356 (2011). 21598397 10.1002/cmdc.201100098

[b28] Gnirss K. *et al.* Cathepsins B and L activate Ebola but not Marburg virus glycoproteins for efficient entry into cell lines and macrophages independent of TMPRSS2 expression. Virology 424, 3–10 (2012). 22222211 10.1016/j.virol.2011.11.031 PMC7111950

[b29] Sanchez A. Analysis of filovirus entry into vero e6 cells, using inhibitors of endocytosis, endosomal acidification, structural integrity, and cathepsin (B and L) activity. J Infect Dis 196 Suppl 2, S251–258 (2007). 17940957 10.1086/520597

[b30] Lemichez E. *et al.* Membrane translocation of diphtheria toxin fragment A exploits early to late endosome trafficking machinery. Mol Microbiol 23, 445–457 (1997). 9044279 10.1111/j.1365-2958.1997.tb02669.x

[b31] Mingo R. M. *et al.* Ebola virus and severe acute respiratory syndrome coronavirus display late cell entry kinetics: evidence that transport to NPC1 + endolysosomes is a rate-defining step. Journal of virology 89, 2931–2943 (2015). 25552710 10.1128/JVI.03398-14 PMC4325712

[b32] Colpitts T. M., Moore A. C., Kolokoltsov A. A. & Davey R. A. Venezuelan equine encephalitis virus infection of mosquito cells requires acidification as well as mosquito homologs of the endocytic proteins Rab5 and Rab7. Virology 369, 78–91 (2007). 17707875 10.1016/j.virol.2007.07.012 PMC2464296

[b33] St Pierre C. A., Leonard D., Corvera S., Kurt-Jones E. A. & Finberg R. W. Antibodies to cell surface proteins redirect intracellular trafficking pathways. Experimental and molecular pathology 91, 723–732 (2011). 21819978 10.1016/j.yexmp.2011.05.011 PMC3315679

[b34] Martinez M. G., Forlenza M. B. & Candurra N. A. Involvement of cellular proteins in Junin arenavirus entry. Biotechnology journal 4, 866–870 (2009). 19548229 10.1002/biot.200800357

[b35] Bernard E. *et al.* Endocytosis of chikungunya virus into mammalian cells: role of clathrin and early endosomal compartments. PLoS One 5, e11479 (2010). 20628602 10.1371/journal.pone.0011479 PMC2900206

[b36] Qa’Dan M., Spyres L. M. & Ballard J. D. pH-induced conformational changes in Clostridium difficile toxin B. Infect Immun 68, 2470–2474 (2000). 10768933 10.1128/iai.68.5.2470-2474.2000 PMC97448

[b37] Johannes L. & Decaudin D. Protein toxins: intracellular trafficking for targeted therapy. Gene Ther 12, 1360–1368 (2005). 15902276 10.1038/sj.gt.3302557

[b38] Jackson M. E., Simpson J. C., Girod A., Pepperkok R., Roberts L. M. & Lord J. M. The KDEL retrieval system is exploited by Pseudomonas exotoxin A, but not by Shiga-like toxin-1, during retrograde transport from the Golgi complex to the endoplasmic reticulum. J Cell Sci 112 (Pt 4) 467–475 (1999). 9914159 10.1242/jcs.112.4.467

[b39] Turcotte S., Letellier J. & Lippe R. Herpes simplex virus type 1 capsids transit by the trans-Golgi network, where viral glycoproteins accumulate independently of capsid egress. Journal of virology 79, 8847–8860 (2005). 15994778 10.1128/JVI.79.14.8847-8860.2005 PMC1168770

[b40] Haspot F. *et al.* Human cytomegalovirus entry into dendritic cells occurs via a macropinocytosis-like pathway in a pH-independent and cholesterol-dependent manner. PLoS One 7, e34795 (2012). 22496863 10.1371/journal.pone.0034795 PMC3322158

[b41] Krzyzaniak M. A., Zumstein M. T., Gerez J. A., Picotti P. & Helenius A. Host cell entry of respiratory syncytial virus involves macropinocytosis followed by proteolytic activation of the F protein. PLoS Pathog 9, e1003309 (2013). 23593008 10.1371/journal.ppat.1003309 PMC3623752

[b42] O’Neill P., Barton V., Ward S. & Chadwick J. 4-Aminoquinolines: Chloroquine, Amodiaquine and Next-Generation Analogues. Springer: Basel AG, (2012).

[b43] Minzi O. M., Rais M., Svensson J. O., Gustafsson L. L. & Ericsson O. High-performance liquid chromatographic method for determination of amodiaquine, chloroquine and their monodesethyl metabolites in biological samples. J Chromatogr B Analyt Technol Biomed Life Sci 783, 473–480 (2003). 10.1016/s1570-0232(02)00727-4 12482490

[b44] Ntale M. *et al.* Field-adapted sampling of whole blood to determine the levels of amodiaquine and its metabolite in children with uncomplicated malaria treated with amodiaquine plus artesunate combination. Malaria journal 8, 52 (2009). 19331684 10.1186/1475-2875-8-52 PMC2678144

[b45] Rijken M. J. *et al.* Pharmacokinetics of amodiaquine and desethylamodiaquine in pregnant and postpartum women with Plasmodium vivax malaria. Antimicrob Agents Chemother 55, 4338–4342 (2011). 21709098 10.1128/AAC.00154-11 PMC3165320

[b46] Lu Q., Wei W., Kowalski P. E., Chang A. C. & Cohen S. N. EST-based genome-wide gene inactivation identifies ARAP3 as a host protein affecting cellular susceptibility to anthrax toxin. Proc Natl Acad Sci USA 101, 17246–17251 (2004). 15569923 10.1073/pnas.0407794101 PMC534609

