# Supplementary material for: Identification of agents effective against multiple toxins and viruses by host-oriented cell targeting
Source: Sci Rep. 2015 Aug 27;5:13476. doi: 10.1038/srep13476 (PMC4550849; doi:10.1038/srep13476)
Supplement: Supplementary Information [file srep13476-s1.pdf]

Identification of agents effective against multiple toxins and viruses by host-oriented cell targeting

Supplemental:

1. Supplemental Figures,
2. Supplemental Tables,
3. Supplemental Experimental Procedures.

Supplemental Figures:

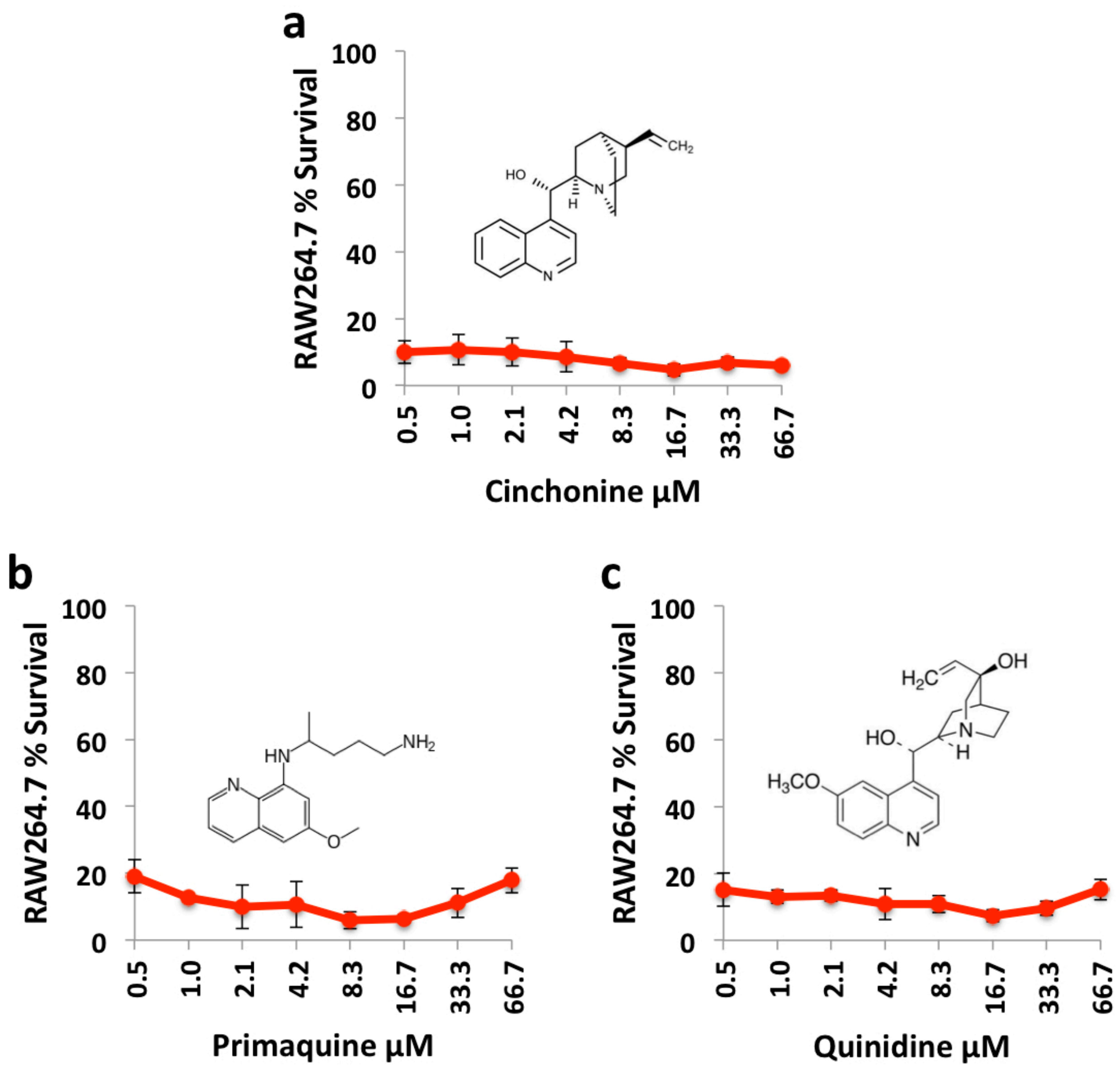

Figure 1: The effect of quinolone anti-malarials on sensitivities of cells treated with anthrax toxins. RAW264.7 cells were pretreated with indicated drugs at shown concentrations for 1 hour, and then treated with anthrax toxins for 6 hours. RAW264.7 survival was measured by MTT assay.

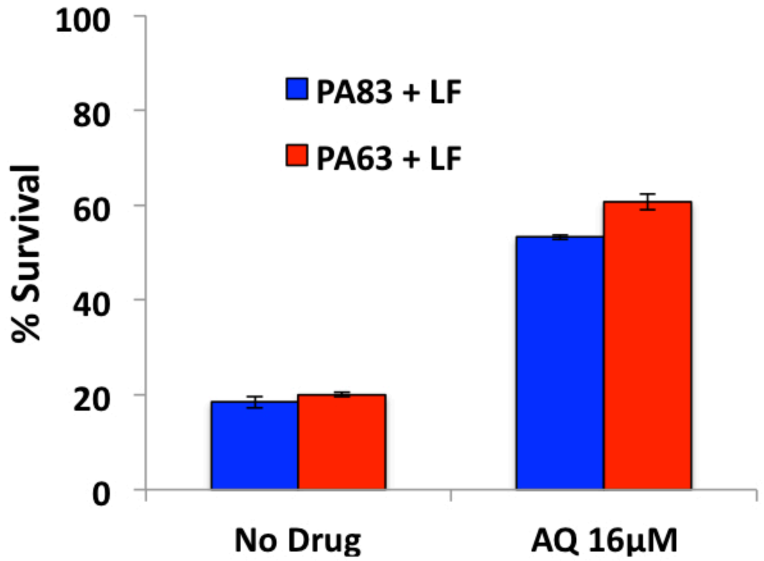

Figure 2: Amodiaquine reduces cellular sensitivity to LF+PA83 and LF+PA63. RAW264.7 cells were pretreated either with DMSO or with AQ for 1 hour, and then treated with 500ng/ml of LF in the presence of 1.5 µg/ml of either of PA83 or PA63 for 6 hours

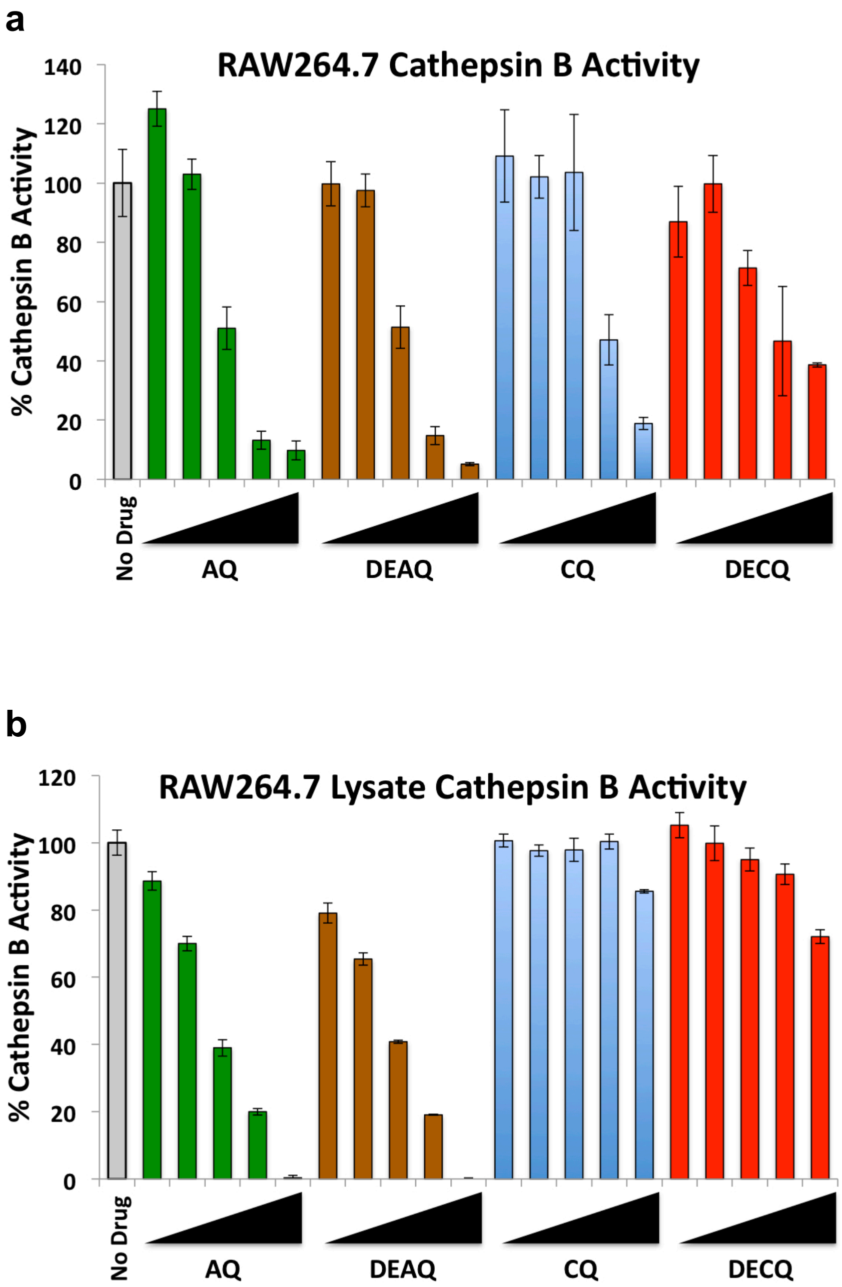

Figure 3: The effect of Amodiaquine, Chloroquine, and their metabolites on the activity of host cathepsin B. FRET assay showing the activity of cathepsin B without drugs, or with addition of AQ, DEAQ, CQ, or DECQ at 4, 8, 16, 33, or 66 μM. RAW264.7 cells were treated with drugs for 1 hour prior to lysis and determination of cathepsin B activity (a), or untreated RAW264 cells were lysed, the lysate was treated with drugs, and then the activity of cathepsin B was assessed (b).

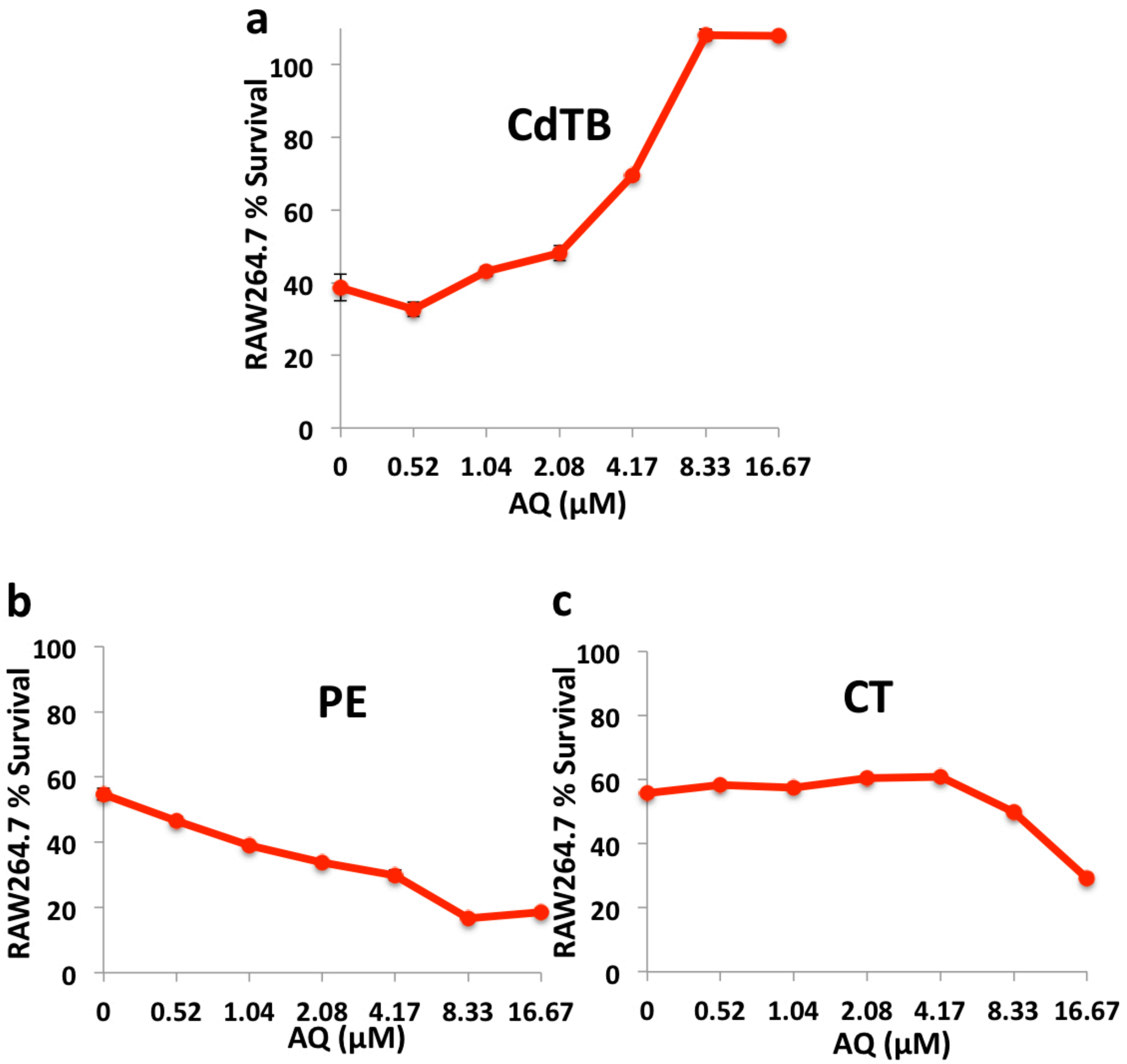

Figure 4: The effect of Amodiaquine on sensitivities of cells treated with bacterial toxins. RAW264.7 cells were pretreated with indicated AQ concentrations for 1 hour, and then treated with (a) 8.3 µg/ml of *Clostridium difficile* toxin B (CdTB), (b) 500 ng/ml of *Pseudomonas aeruginosa* Exotoxin A (PE), or (c) 500 ng/ml of Cholera toxin (CT) for 6 hours. RAW264.7 survival was measured by MTT assay.

Supplemental Tables:

Pathogen	Drug, hours	EC50 µM	SD µM	CC50 µM	SI50
EBOV	AQ, 48h	2.6	0.19	30	11.4
EBOV	DEAQ, 48h	2.1	0.48	30	14.4
EBOV	CQ, 48h	2.5	0.88	70	28.0
EBOV	DECQ, 48h	3.2	0.62	70	22.1

Table 1: Amodiaquine, Chloroquine and theirmetabolites inhibit Ebola virus in HFF-1 cells. The effect of AQ, CQ, and its metabolites, DEAQ and DECQ, on the pathogenicity of EBOV in HFF-1 cells. The ability of drugs to reduce the abundance of Ebola virus (EBOV) in host cells was measured in cells by fluorescent microscopy. The 50% effective (EC_{50}, virus-inhibitory) concentrations and 50% cytotoxic (CC_{50}, cell-inhibitory) concentrations were determined. CC_{50} divided by EC_{50} indicate the selectivity index (SI) value.

Pathogen	Drug	EC50 µM	SD µM	CC50 µM	SI50
CHIV	AQ	18.4	0.56	>50	>2
CHIV	DEAQ	17.3	0.64	>50	>2.9
JUNV	AQ	16.9	1.92	50	3.0
JUNV	DEAQ	13.8	2.02	40	2.9

Table 2: Amodiaquine and its metabolite inhibits Chikungunya (CHIV) and Junin (JUNV) viruses in host cells. The effect of AQ and DEAQ on the pathogenicity of EBOV in HFF-1cells. The ability of drugs to reduce the abundance of viruses in host cells was measured in cells by fluorescent microscopy. The 50% effective (EC_{50}, virus-inhibitory) concentrations and 50% cytotoxic (CC_{50}, cell-inhibitory) concentrations were determined. CC_{50} divided by EC_{50} indicate the selectivity index (SI) value.

Supplemental Materials and Methods:

Screening Assays for Ebola virus (EBOV, Strain Kikwit).

High Content Imaging virus infection assay

Cell Culture and infection

HeLa cells (ATCC) were cultured for 3 days in T175 or T225 (Corning) flasks in culture media containing Minimum Essential Medium (MEM, Corning Cellgro) supplemented with 10% Fetal Bovine Serum (Hyclone), 1% L-Glutammine (Hyclone), 10 mM Hepes 7.0-7.6 (Sigma), 1% non-essential amino acids (Sigma). Cells were lifted using Trypsin-EDTA (Sigma, T3974) and 2,000 cells/well were plated in 35µl of culture media into imaging 384 well assay plates (Aurora 384, IQ-EB, 384 IQ-EB/NB, 200mclear, #1052-11130) and incubated for about 20h before compound treatment.

Compound treatment

Treatment of the cells with test and control compounds was done 2 h prior infection. EC50 determination for test compound done with 2 fold step at least for 10 doses starting from highest concentration of 100uM (with stock concentration of 10mM) using HP D-300 digital dispenser. Each dose dispensed directly from the concentrated stock with highest volume of 500nL. Concentration of DMSO in all wells was normalized to 1%. Cells were pre-treated with serially diluted compounds for 2 hours before infection. Each dose were repeated 4 times on one plate (n=4).

Infection

Infection was done using Ebola virus (Kikwit) MOI = 0.5 (calculated for 4,000 cells/well, assuming one complete round of replication of HeLa cells at 15±2 hrs after cell seeding) and 10ul of virus dilution was dispensed in each well except column 2, representing "no infection control". Cells were incubated with the virus for 24 or 48h. Infection was terminated by fixing samples in formalin solution.

Immuno-staining assay

Immune-staining was used to visualize infected cells. Cells were treated with anti-GP specific monoclonal antibody (6D8) (1 to 1000 dilution) followed by anti-mouse IgG conjugated with Dylight488 (Thermo) (1 to 1000 dilution) in blocking buffer containing 3% BSA in PBS. Nuclei were stained with Draq5 (Biostatus) diluted in PBS buffer.

Image and Data analysis.

Images were acquired on the Opera imaging instrument (Perkin Elmer) using 10x Air objective and four images/well were acquired. Signal from anti-virus staining was detected at 488nm emission wavelength and Nuclei at 640nm.
Image analysis was performed using PE Acapella algorithms. Several well-based output parameters were measured including: Number of Objects (Nuclei) and Positive Virus Number of Objects (Ave of Intensity for virus specific signal). The % of Infected cells were calculated by Acapella directly as

$$\% \, Infected \; cells \; = \frac{Positive \; Virus \; Number \; of \; Objects \; (s)}{Number \; of \; objects \; (s)} * 100$$

% infection rate was analyzed for each plate using Median of % Positive virus cells (for all Neutral control wells that are infected and treated with DMSO only; S-sample

Data analysis

Data normalization

% Inhibition. Data normalization was done using GeneData Explorer software. Data was normalized on the plate based level converting the %Virus positive cells associated with each wells into % Activity = % of inhibition of infection (or % Inhibition or % INH). Control wells were indicated as follows:

NC =Neutral control; Infection + DMSO (or minimal activity of inhibitor)
BC = Blank control; No infection (or maximum inhibition of viral infection possible). Median of the values is used for all controls.

$$\% \, Inhibition = \frac{Median \; \%Virus \; positive \; (NC) - \; \%Virus \; positive (S)}{Median \; \%Virus \; positive (NC) - Median \; \%Virus \; positive (BC)} * 100\%$$

% Viability. Number of objects represented the amount of cells in each treated well and was also used as indication of the cyto-toxic or cyto-static effect. The number of objects was converted by GeneData Condeseo to % Viability for with a compound dose response.

$$\%Viability = \frac{Nuclei \; Nuber \; Sample}{MedianNulei \; number \; (NC)} * 100\%$$

Dose response curve fitting analysis

Analysis of dose response curve for % Inhibition and % Viability was used to determine EC_{50} and CC_{50}, and was applying GeneData Condoseo software with Levenberg-Marquardt algorithm (LMA) for curve fitting strategy. The Curve-fitting applied validity criteria, such aschi^2, SE logEC_{50}, minimal number of valid data points, to indicate if curve fitting converging was successful and indicated in table with results. R^2value quantifies goodness of fit. Fitting strategy was considered acceptable if it gave conversion with R^2>0.8.

Screening Assays for Venezuelan equine encephalitis virus (VEEV, Strain TC-83).

Primary cytopathic effect (CPE) reduction assay. Four-concentration CPE inhibition assays are performed. Confluent or near-confluent cell culture monolayers in 96-well disposable microplates are prepared. Cells are maintained in MEM or DMEM supplemented with FBS as required for each cell line. For antiviral assays the same medium is used but with FBS reduced to 2% or less and supplemented with 50 µg/ml gentamicin. The test compound is prepared at four $\log_{10}$ final concentrations, usually

0.1, 1.0, 10, and 100 µg/ml or µM. The virus control and cell control wells are on every microplate. In parallel, a known active drug is tested as a positive control drug using the same method as is applied for test compounds. The positive control is tested with each test run. The assay is set up by first removing growth media from the 96-well plates of cells. Then the test compound is applied in 0.1 ml volume to wells at 2X concentration. Virus, normally at <100 50% cell culture infectious doses (CCID50) in 0.1 ml volume, is placed in those wells designated for virus infection. Medium devoid of virus is placed in toxicity control wells and cell control wells. Virus control wells are treated similarly with virus. Plates are incubated at 37°C with 5% CO_2 until maximum CPE is observed in virus control wells. The plates are then stained with 0.011% neutral red for approximately two hours at 37°C in a 5% CO_2 incubator. The neutral red medium is removed by complete aspiration, and the cells may be rinsed 1X with phosphate buffered solution (PBS) to remove residual dye. The PBS is completely removed and the incorporated neutral red is eluted with 50% Sorensen's citrate buffer/50% ethanol (pH 4.2) for at least 30 minutes. Neutral red dye penetrates into living cells, thus, the more intense the red color, the larger the number of viable cells present in the wells. The dye content in each well is quantified using a 96-well spectrophotometer at 540 nm wavelength. The dye content in each set of wells is converted to a percentage of dye present in untreated control wells using a Microsoft Excel computer-based spreadsheet. The 50% effective (EC_{50}, virus-inhibitory) concentrations and 50% cytotoxic (CC_{50}, cell-inhibitory) concentrations are then calculated by linear regression analysis. The quotient of CC_{50} divided by EC_{50} gives the selectivity index (SI) value.

Screening Assays for Herpes Simplex Virus 1 (HSV-1, Strain E-377) and Human Cytomegalovirus (CMV, Strain AD169).

Human foreskin fibroblast (HFF) cells were prepared from human foreskin tissue. The tissue was incubated at 4°C for 4 h in Clinical Medium and then placed in phosphate buffered saline (PBS) to remove the red blood cells, and resuspended in trypsin/EDTA solution. The tissue suspension was incubated at 37°C and gently agitated to disperse the cells, which were collected by centrifugation. Cells were resuspended in 4 ml Clinical Medium and placed in a flask and incubated at 37°C in a humidified CO_2 incubator for 24 h. The media was then replaced with fresh Clinical Medium and the cell growth was monitored daily until a confluent monolayer has formed. The HFF cells were then expanded through serial passages in standard growth medium of MEM with Earl's salts supplemented with 10% FBS and antibiotics. The cells were passaged routinely and used for assays at or below passage 10.

Primary Cytopathic Effect (CPE) Reduction Assay. Low passage (3-10) HFF cells were trypsinized, counted, and seeded into 96 well tissue culture plates in 0.1 ml of MEM supplemented with 10% FBS. The cells were then incubated for 24 h at 37°C. The media was then removed and 100 µl of MEM containing 2% FBS was added to all but the first row. In the first row, 125 µl of media containing the experimental drug was added in triplicate wells. Media alone was added to both cell and virus control wells. The drug in the first row of wells was then diluted serially 1:5 throughout the remaining. The plates were then incubated for 60 min and 100 µl of a virus suspension was added to each well, excluding cell control wells which received 100 µl of MEM. The plates were

then incubated at 37^oC in a CO_2 incubator for three days for HSV-1, or 14 d for CMV. After the incubation period, media was aspirated and the cells stained with crystal violet in formalin for 4h. The stain was then removed and the plates were rinsed until all excess stain was removed. The plates were allowed to dry for 24 h and the amount of CPE in each row determined using a BioTek Multiplate Autoreader. EC_{50} and CC_{50} values were determined by comparing drug treated and untreated cells using a computer program.

Screening Assays for Rabies (Strain Flury).

Confluent BHK-21 cells were prepared in T-150 flask. Cells were Trypsinized cells and made as cell suspensions. 50 μl of $5x10^5$ cells/ml cell suspension (25,000 cells/well) were added into each well of the 96 well plates, except row H. 2X concentration of antivirals were made (Isoprinosine and test drug(s)). 6 concentration points are done. 100μl per well that will be tested for effective concentration (drug wells) and cytotoxic concentration (tox wells) were added. The rabies virus was diluted 1:1000. 50μl was added per well in the drug wells and virus control wells. This gave a final dilution of 1:4000. The final volume was 200μl. Additional media was added to fill up wells, ie: Tox wells were 100μl 2x drug, 50μl cells (5e5 cells/ml), and 50μl media. Plates were covered and incubated (37°C; 5 % CO_2) for 5 days. After 5 days the Promega CellTiter-Glo Luminescent Cell Viability Assay was run using the Fluoroskan FL to scan for luminescence.

Screening Assays for Poliovirus 3

Principal Viruses and Cells:
Poliovirus: WM3, Cells: LLC-MK2 clone 7.1

CellTiter 96 (Cytopathic effect/Toxicity). The primary screen is a cytopathic effect (CPE) reduction assay. Briefly, 96-well cultures of cells are infected with virus in the presence of test compounds and incubated for 4-7 days (depending on the specific virus/cells). Each virus is pre-titered such that control wells exhibit approximately 95% loss of cell viability due to virus replication. Therefore, antiviral effect, or cytoprotection, is observed when compounds prevent virus replication. Each assay plate contains cell control wells (cells only), virus control wells (cells plus virus), compound toxicity control wells (cells plus compound only), compound colorimetric control wells (compound only, no cells or virus), as well as experimental wells (compound plus cells plus virus). Cytoprotection and compound cytotoxicity are assessed by MTS (CellTiter®96 Reagent, Promega, Madison WI) dye reduction. The % reduction in viral CPE (antiviral activity) and % cell viability (cytotoxicity) are determined and reported.

Screening Assays for Respiratory syncytial virus and SARS CoV

Principal Viruses and Cells:
Respiratory syncytial virus (RSV): Strain A-2 in Hep2 cells
SARS CoV: Strain Urbani in Vero76 cells

CellTiter-Glo (Cytopathic effect/Toxicity). The antiviral cytoprotection assays examine the effects of compounds at designated dose-response concentrations in specific cell types to test the efficacy of the compounds in preventing the virus-induced cytopathic effect. Ribavirin is included as a positive control drug for influenza and RSV, while calpain IV inhibitor is used for SARS antiviral assays. Subconfluent cultures of cells are plated into 96-well plates for the analysis of cell viability (cytotoxicity) and antiviral activity (CPE). For the standard assay, drugs are added to the cells 24 hours later. The CPE wells also received 100 tissue culture infectious doses (100 $TCID_{50}$s) of titered virus. 72 hours later the cell viability will be determined. Measurement of viral-induced CPE is based on quantitation of ATP, an indicator of metabolically active cells. The CPE assay employs a commercially available CellTiter-Glo® Luminescent Cell Viability Kit (Promega, Madison, WI), and is a reliable method for determining cytotoxicity and cell proliferation in culture. The procedure involves adding the single reagent (CellTiter-Glo Reagent) directly to previously cultured, subconfluent cells in media. This induces cell lysis and the production of a bioluminescent signal (half-life greater than 5 hours, depending on the cell type) that is proportional to the amount of ATP present (which is a biomarker for viability).
